# Single Cell Analysis of Lymph Node Tissue from HIV-1 Infected Patients Reveals that the Majority of CD4^+^ T-cells Contain One HIV-1 DNA Molecule

**DOI:** 10.1371/journal.ppat.1003432

**Published:** 2013-06-20

**Authors:** Lina Josefsson, Sarah Palmer, Nuno R. Faria, Philippe Lemey, Joseph Casazza, David Ambrozak, Mary Kearney, Wei Shao, Shyamasundaran Kottilil, Michael Sneller, John Mellors, John M. Coffin, Frank Maldarelli

**Affiliations:** 1 Department of Microbiology, Tumor and Cell-biology, Karolinska Institutet, Solna, Sweden; 2 Department of Diagnostics and Vaccinology, Swedish Institute for Communicable Disease Control, Solna, Sweden; 3 HIV Drug Resistance Program, National Cancer Institute, National Institutes of Health, Frederick, Maryland, United States of America; 4 Department of Microbiology and Immunology, Rega Institute, Katholieke Universiteit Leuven, Leuven, Belgium; 5 Vaccine Research Center, National Institute of Allergy and Infectious Diseases, National Institutes of Health, Bethesda, Maryland, United States of America; 6 Laboratory of Immunoregulation, National Institute of Allergy and Infectious Diseases, National Institutes of Health, Bethesda, Maryland, United States of America; 7 Department of Medicine, Division of Infectious Diseases University of Pittsburgh, Pittsburgh, Pennsylvania, United States of America; 8 Department of Molecular Biology and Microbiology, Tufts University, Boston, Massachusetts, United States of America; University of Massachusetts Medical School, United States of America

## Abstract

Genetic recombination contributes to the diversity of human immunodeficiency virus (HIV-1). Productive HIV-1 recombination is, however, dependent on both the number of HIV-1 genomes per infected cell and the genetic relationship between these viral genomes. A detailed analysis of the number of proviruses and their genetic relationship in infected cells isolated from peripheral blood and tissue compartments is therefore important for understanding HIV-1 recombination, genetic diversity and the dynamics of HIV-1 infection. To address these issues, we used a previously developed single-cell sequencing technique to quantify and genetically characterize individual HIV-1 DNA molecules from single cells in lymph node tissue and peripheral blood. Analysis of memory and naïve CD4^+^ T cells from paired lymph node and peripheral blood samples from five untreated chronically infected patients revealed that the majority of these HIV-1-infected cells (>90%) contain only one copy of HIV-1 DNA, implying a limited potential for productive recombination in virus produced by these cells in these two compartments. Phylogenetic analysis revealed genetic similarity of HIV-1 DNA in memory and naïve CD4^+^ T-cells from lymph node, peripheral blood and HIV-1 RNA from plasma, implying exchange of virus and/or infected cells between these compartments in untreated chronic infection.

## Author Summary

One of the greatest challenges facing treatment and vaccine development for human immunodeficiency virus (HIV-1) is the genetic diversity of the virus. One of the main factors contributing to HIV-1 diversity is recombination between two genetically different viral RNA genomes that enter a cell in the same virion. Such heterozygous virions can only arise from cells that contain two or more genetically distinct HIV-1 proviruses. Therefore, the amount of productive HIV-1 recombination in infected individuals is dependent on the number of multiple infected cells and the genetic relationship of the proviruses they contain. In this work we use a recently developed assay, single-cell sequencing, to analyze the number and genetic makeup of HIV-1 DNA molecules in single infected cells. We used this assay to analyze memory and naïve CD4^+^ T cells from lymph node tissue and peripheral blood sampled from five chronically untreated HIV-1 infected individuals. Our results revealed that <10% of infected memory and naïve T-cells from either the lymph node tissue or peripheral blood are multiply infected, a number far below earlier estimates. In addition, we demonstrate a similar genetic composition of HIV-1 in lymph node tissue, peripheral blood and plasma during untreated chronic HIV-1 infection.

## Introduction

The genetic diversity of human immunodeficiency virus (HIV-1) allows the virus to develop resistance to antiviral therapy and escape immune pressure. Several different mechanisms contribute to genetic diversity including rapid, high-level virus turnover (ca. 10^8^–10^9^ cells are infected and die every day), nucleotide misincorporation during replication of the HIV-1 genome, and recombination [1]–[3].

HIV-1 recombination, which generates new viral variants through a process of genetic exchange, is initiated when a cell is infected by genetically distinct HIV-1 variants and two RNAs transcribed from the different proviruses are co-packaged into a virion. Subsequent infection of new host cells proceeds with reverse transcription, template switching of reverse transcriptase (RT) between the two genetically different genomic RNAs, leading to a recombinant genome that is genetically different from either of the two parental variants. Therefore, an essential and rate limiting step in the process of productive HIV-1 recombination is the co-infection of cells by two or more genetically distinct HIV-1 variants [4], [5].

To investigate the numbers of cells co-infected by different HIV-1 variants in peripheral blood, we developed the single-cell sequencing (SCS) assay, which allows for the analysis of HIV-1 DNA molecules at a single cell level. Using this assay, we found that the majority of CD4^+^ T-cells (>90%) from the peripheral blood of untreated HIV-1-infected patients contain a single HIV-1 DNA molecule [6]. In contrast, other studies reported that CD4^+^ cells from the spleen are multiply infected by HIV-1 *in vivo* [7]–[9]. These isolated spleen cells were found to harbor one to eight (with a mean of 3.2) genetically diverse proviruses per cell. The discrepancy between our study and these studies of cells isolated from the spleen may be attributable to the source of cells; peripheral blood versus lymphoid tissue. Only 2% of the total amount of lymphocytes are found in the peripheral circulation [10], with the remainder distributed throughout the body, especially in lymphoid organs — such as lymph nodes and the gut associated lymphoid tissue (GALT) where most HIV-1 infection takes place [11]–[14] and where cells are tightly packed, facilitating cell to cell HIV transmission. Therefore, increased frequency of HIV-1 infection and cell exposure to HIV-1 virions may lead to more multiply infected cells in lymphoid tissue than in peripheral blood, which may have substantial clinical consequences. Recent data from Sigal et al. suggest that cell to cell transmission of individual drug resistant mutants in the setting of suboptimal antiretroviral therapy can yield virions capable of generating recombinant viruses encoding multiple drug resistance mutations, thereby rapidly limiting the effectiveness of antiretroviral drugs [15]. An increased frequency of HIV-1 infection has been shown in different tissue compartments and in different cellular subsets. CD4^+^ T-cells are the major target for HIV-1 infection and both naïve and memory CD4^+^ T-cells have been shown to be permissive, with most viral DNA detected in CD4^+^ T-cells that express the memory marker CD45RO [16]–[19].

To investigate the viral genetic composition, frequency of infection and number of multiply infected cells from different tissue compartments and cellular populations, we applied the recently developed single-cell sequencing assay to analyze single infected naïve and memory CD4^+^ T-cells sorted from paired lymph node tissue and peripheral blood samples. We determined that the majority of infected CD4^+^ T-cells (>90%) located in lymph node tissue and peripheral blood contain only one HIV-1 DNA molecule, implying limited potential for productive recombination in viruses produced by these cells. Furthermore, we show that viral sequences isolated from memory and naïve T-cells from lymph node tissue and peripheral blood are similar to each other and to HIV-1 RNA from contemporaneous plasma from these patients, indicating an exchange of genetic material between these compartments in untreated chronic HIV-1 infection.

## Results

### The majority of CD4^+^ T-cells isolated from lymph node tissue and peripheral blood contain one HIV-1 DNA molecule

The rate of HIV-1 recombination in infected individuals is dependent on the number of cells infected with two or more genetically distinct variants. To investigate the number and genetic makeup of proviruses in CD4^+^ T-cells residing within lymph node tissue and peripheral blood, we analyzed memory and naïve T-cells from these two compartments in five HIV-1 infected individuals using single-cell sequencing. All five patients were chronically infected with HIV-1 (Fiebig stage VI) and were not receiving antiretroviral therapy. All were male, relatively young (median age 30 y) and had a broad range of CD4^+^ T-cell concentrations and viral RNA levels (**Table 1**). All of the lymph nodes had evidence of typical HIV-1 infection that included florid follicular hyperplasia.

**Table 1:** Patient demographics.

Patient	Age	Sex	Race/Ethinicity	HIV Risk	Duration of Infection (y)^1^	HIV Plasma RNA (bDNA. copies/ml)	CD4 (cells/µl)
1	21.5	M	White/Latino	Heterosexual	0.7	35576	758
2	29.9	M	Black	MSM	7.9	9991	935
3	29.7	M	Black	Heterosexual	<1^2^	293361	220
4	33.2	M	White/latino	MSM	11.9	3618	121
5	40.6	M	Black	Heterosexual	7	6195	484

1 Estimated from first positive western blot.

2 Participant had negative ELISA 1 year prior to entry.

The single-cell sequencing assay was recently published by our group and allows us to both quantify and genetically characterize the virus found in single infected cells [6]. Pools of cells, each containing <<1 infected cell, are lysed and distributed across 10 wells per row in a total of 8 rows per 96 well PCR plate (for detailed information how cell concentrations with << 1 infected cell were obtained please see [6] and the Material and Methods section below). During this analysis of cells from peripheral blood and lymph node tissue the number of infected cells per lane of 10 wells ranged from 0.03 to 0.4. PCR amplification and sequencing of the DNA in each well allows enumeration and analysis of the genetic relationship of viral DNA molecules in each infected cell. Using Poisson statistical methods, we also determined the predicted number of multiply infected cells (Table 2, parentheses), and whether the observed number of multiply infected cells exceeded predicted.

**Table 2:** Frequency of infection.

1	2	3	4	5	6	7	8	9	10	11	12	13
	Observed number of HIV DNA molecules						Frequecy of multiple infection		Method 1†		Method 2‡	
Patient	Zero	One	Two	Three	Four	P-value for null hypothesis of no multiple infection	Upper 95% confidence bound	Most consistent with observed no. of multiple HIV DNA molecules (%)	Cells/HIV DNA	95% CI	Cells/HIV DNA	95% CI
**Lymphoid Tissue - Memory T-cells**												
1	215 (211.5)*	60 (64.4)	10 (10.7)	2 (1.2)	1 (0.1)	0.42	26.8%	1.5%	160	130, 199	197	157, 252
2	186 (183.2)	31 (36.8)	7 (3.7)	0 (0.2)	0 (0)	0.10	31.8%	8.2%	498	372, 682	589	430, 833
3	151 (145.0)	30 (39.2)	8 (6.8)	2 (0.9)	1 (0.1)	0.16	40.5%	8.2%	69	53, 91	94	69, 130
4	160 (156.1)	25 (32.1)	6 (3.5)	1 (0.3)	0 (0)	0.09	37.2%	9.9%	384	282, 537	480	340, 702
5	151 (144.9)	31 (40.8)	7 (5.7)	3 (0.5)	0 (0)	0.10	37.9%	9.0%	213	164, 284	281	207, 391
**Combining all Lymphoid Tissue - Memory T-cells**												
	863 (840.8)	177 (213.3)	38 (30.4)	8 (3.2)	2 (0.3)	**0.01**	26.3%	6.6%	214**		271**	
**Lymphoid Tissue- Naive T-cells**												
1	85 (85.6)	11 (9.8)	0 (0.6)	0 (0)	0 (0)	1.00	23.8%	0.0%	8727	4878, 17482	8727	4878, 17482
2	59 (57.4)	3 (6.2)	2 (0.4)	0 (0)	0 (0)	0.06	81.1%	25.9%	6857	3328, 17055	9600	4114, 29565
3	127 (123.1)	26 (32.3)	5 (4.2)	2 (0.4)	0 (0)	0.18	36.2%	7.3%	381	282, 528	485	345, 704
4	30 (29.6)	9 (8.9)	0 (1.3)	1 (0.1)	0 (0)	0.78	39.4%	0.0%	1000	573, 1935	1200	653, 2502
5	119 (117.4)	14 (17.2)	3 (1.3)	0 (0.1)	0 (0)	0.15	39.6%	9.6%	3320	2150, 5435	3906	2440, 6705
**Combining all Lymphoid Tissue - Naive T-cells**												
	420 (413.1)	63 (74.4)	10 (7.8)	3 (0.6)	0 (0)	**0.08**	25.8%	6.1%	2376		2857	
**Peripheral Blood -Memory T-cells**												
1	138 (138.1)	6 (5.7)	0 (0.1)	0 (0)	0 (0)	1.00	39.3%	0.0%	4000	1838, 10899	4000	1838, 10899
2	206 (205.1)	23 (25.0)	3 (1.9)	0 (0.1)	0 (0)	0.32	27.2%	4.0%	1738	1210, 2595	1938	1323, 2967
3	75 (74.8)	18 (18.7)	3 (2.3)	0 (0.2)	0 (0)	0.47	32.9%	2.5%	120	81, 187	137	90, 221
4	26 (23.4)	3 (7.3)	2 (1.1)	1 (0.1)	0 (0)	0.13	84.7%	24.0%	320	174, 667	533	245, 1453
5	79 (77.1)	14 (16.9)	2 (1.8)	1 (0.1)	0 (0)	0.32	39.6%	6.0%	457	299, 738	565	353, 969
**Combining all Peripheral Blood - Memory T-cells**												
	524 (518.5)	64 (73.6)	10 (7.2)	2 (0.5)	0 (0)	**0.10**	24.3%	5.6%	657		796	
**Peripheral Blood -Naive T-cells**												
1												
2	20	0	0	0	0	(n/a)	n/a	(n/a)	n/a	n/a	n/a	n/a
3												
4												
5	35 (35.1)	11 (11.0)	2 (1.7)	0 (0.2)	0 (0)	0.58	41.0%	0.8%	1600	970, 2858	1846	1080, 3467

† Method 1: Assuming no multiple infection.

‡ Method 2: Assuming maximal multiple infection.

* Poisson predicted values in parentheses.

** Geometric mean of infection values.

Using this assay we isolated and analyzed memory CD4^+^ T-cells from both lymph node tissue and peripheral blood and found that the majority (>90%) of the infected cells contained only one HIV-1 DNA molecule (**Table 2** **, column 9**). Naïve T-cells isolated from lymph node tissue had a similarly high proportion of singly infected cells. In four of the five patients (2–5) we found evidence for multiple infection (multiple HIV-1 DNA molecules in at least one row of 10 wells) of memory CD4^+^ T-cells from both lymph node tissue and peripheral blood and naïve T- cells from lymph node tissue. Also, patient 5 showed evidence of multiple infection in naïve T-cells isolated from peripheral blood. In each case, however, the number of rows containing more than one HIV-1 DNA molecule corresponded closely to Poisson predicted values under the assumption that no multiple infection was present (**Table 2** **, columns 2–6**, values in parenthesis are Poisson predicted values). This result indicates that the detection of multiple HIV-1 DNA molecules in the same row could be due to several singly infected cells being analyzed in the same row rather than presence of one cell containing multiple HIV-1 DNA molecules. In addition, for each patient individually, there was insufficient statistical support to reject the hypothesis that there was no multiple infection present (p values ranging from 0.06 to 1, **Table 2** **, column 7**). Combining all the data for all the patients, we did, however, find statistical support for multiple infection in memory CD4^+^ T-cells isolated from lymph node tissue (p = 0.01). The estimated frequencies of multiple infection in naïve CD4^+^ T-cells isolated from the same cellular compartment (p = 0.08) or memory CD4^+^ T-cells from peripheral blood (p = 0.1) did not reach statistical significance, although the low p-values suggest that some double infection may have occurred as well.

Even though we cannot definitively determine the frequency of multiple infection in the different cell types from the data in Table 2, we can use these data to establish confidence limits for the frequency of multiple infection. If we conservatively assume that all rows with multiple HIV-1 DNA molecules are the result of multiply infected cells, we conclude with an upper confidence level of 95% that no more than 26.3% (26.8–40.5%) and 25.8% (23.8–81.1%) of the memory and naïve CD4^+^ T-cells, respectively, isolated from lymph node tissue are multiply infected. Similarly, the percent of multiply infected memory CD4^+^ T-cells from peripheral blood is no greater than 24.3% (27.2–84.7%) (**Table 2** **, column 8**). However, when simulations were conducted to estimate the rate of multiple infection most consistent with the observed distribution of HIV-1 DNA molecules, we found that the percent of multiply infected cells is no greater than 7% in any of the cellular subsets analyzed (**Table 2** **, column 9**). Interestingly, frequencies of multiply infected cells were similar across most patients despite 100-fold differences in plasma viremia.

These results are in agreement with our recent data [6] and demonstrate that the majority of CD4^+^ T-cells isolated from both lymph node tissue and peripheral blood contain only one HIV-1 DNA molecule.

### Infection frequencies of CD4^+^ T-cells isolated from lymph node and peripheral blood

**Figure 1:**
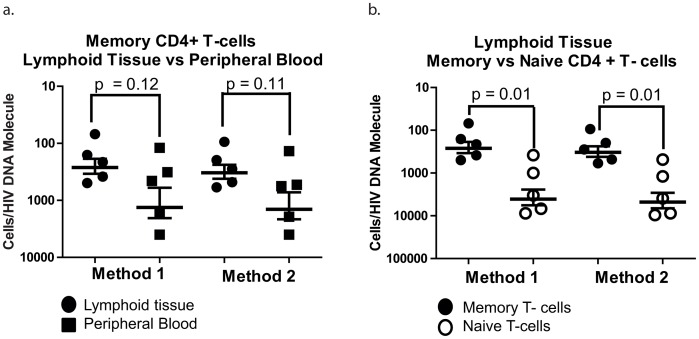
Frequency of HIV-1 infection of memory and naïve cells in blood and lymph nodes. (a) Frequency of infection of memory CD4^+^ T-cells isolated from lymph node tissue vs memory CD4^+^ T-cells isolated from peripheral blood. (b) Frequency of infection of memory CD4^+^ T-cells vs naïve CD4^+^ T-cells isolated from lymph node tissue. Frequency of infection for each patient and cell type was calculated using two methods. Method 1 assumes that each observed amplicon is assumed to derive from a unique infected cell (i.e., no multiple infection); Method 2 assumes that each row with two or more amplicons represents a multiply infected cell (assuming a maximal rate of multiple infection). Frequency of infection is shown as total number of cells per HIV-1 DNA molecule. Differences in frequency of infection between the different cellular compartments and cell types (here represented as p-values in the figure) are based on paired *t* tests of log-transformed frequencies of infection.

Using the single-cell sequencing assay we analyzed the frequency of infection in memory and naïve CD4^+^ T-cells from both lymph node tissue and peripheral blood. The frequency of infection for each cell type was based on the total number of cells analyzed divided by the number of infected cells, as determined by the number of HIV-1 DNA molecules detected in each row of 10 wells. This approach yields precise measurements of infection frequency, permitting direct comparisons of frequency of infection in various cell subsets and anatomic compartments. Due to the design of the single-cell sequencing assay, where multiple DNA molecules in one row of 10 wells could have been derived from either singly or multiply infected cells, we used two methods to calculate the frequency of infection. For method 1, all multiple HIV-1 DNA molecules found in one row were assumed to derive from singly infected cells (no occurrence of multiple infection), yielding a higher frequency of infection than method 2 where all multiple HIV-1 DNA molecules found in one row were assumed to derive from multiply infected cells (maximal occurrence of multiple infection), yielding a lower frequency of infection (**Table 2** **, columns 10–13**). These calculations yielded a geometric mean of 214–271 cells/HIV-1 DNA molecule in memory CD4+ T-cells from lymph node tissue and a geometric mean of 657–796 cells/HIV-1 DNA molecule in memory CD4+ T-cells isolated from peripheral blood (patients 1–5, **Table 2** **, “patients combined” columns 10–13**). In four of the five patients (patients 1, 2, 3 and 5) the HIV-1 infection frequency of memory CD4^+^ T-cells from lymph node tissue was 2–17 times higher than the infection frequency of memory CD4^+^ T-cells from peripheral blood. When memory CD4^+^ T-cells sorted from lymph node tissue and peripheral blood for all five patients were compared, however, the 3-fold difference in infection frequencies of the cells from these two separate compartments was not statistically significant (p = 0.12 and 0.11 (Method 1 and 2 respectively, paired *t* test, **Fig. 1a**). The same result was also obtained when data from patient 4 (who had similar infection frequencies in CD4^+^ T-cells from lymph node tissue and peripheral blood) were removed from the analysis. Moreover, higher infection frequencies in memory T-cells from peripheral blood were positively correlated with plasma RNA levels, consistent with previous observations [16]. We also detected a strong positive correlation between frequencies of infected memory cells in lymphoid tissue and plasma viral RNA level (r^2^ = 0.77 and 0.79, method 1 and 2, respectively).

We also sorted naïve CD4^+^ T-cells from lymph node tissue from all five patients and the infection frequency of these cells ranged from 2376–2857 cells/HIV-1 DNA molecule (geometric mean of patients 1–5, **Table 2** **, columns 10–13**). The infection frequency of naïve CD4^+^ T-cells was significantly lower (12-fold) than the infection frequency of memory CD4^+^ T-cells sorted from lymph node tissue (p = 0.01 and 0.01, method 1 and 2 respectively, paired *t* test, **Fig. 1b**). Naïve CD4^+^ T-cells could only be isolated from peripheral blood from two of the five patients (patients 2 and 5) and those cells from patient 2 did not contain any detectable HIV-1 DNA (0 copies per 10000 naïve T-cells analyzed) indicating an infection frequency of >10000 cells/HIV-1 DNA molecule. The infection frequency of naïve CD4^+^ T-cells isolated from peripheral blood of patient 5 ranged from 1600–1846 cells/HIV-1 DNA molecule, similar to the overall frequency of infection of naïve CD4^+^ T-cells in lymph node tissue (2376–2857 cells/HIV-1 DNA molecule). In addition, we estimated the contribution that each of the infected cells makes to the total HIV-1 reservoir in CD4^+^ T-cells in peripheral blood and lymph node tissue. In samples from lymph node tissue, we found that the mean % contribution was 93.8 and 6.2% in memory and naïve T-cells, respectively. Also in peripheral blood memory T-cells were the major component of the HIV-1 reservoir (mean % contribution was 70.9 and 29.1% in memory and naïve CD4^+^ T-cells respectively, patients 3 and 5). To directly compare the distribution of HIV-1 infected cells in the combined peripheral blood and lymph node compartments, we calculated the total levels of HIV-1 infected cells in peripheral blood and in the lymphoid tissue (extrapolating from our lymph node data and excluding spleen and GALT). In general, total levels of HIV-1 infected cells in lymphoid tissue exceeded peripheral blood although the proportions varied; 80–97% of all infected memory cells and 92–100% of all infected naïve cells are present in lymph nodes

### Phylogenetically similar sequences from intracellular HIV-1 DNA and contemporaneous plasma RNA

To evaluate the genetic relationship between the HIV-1 DNA sequences that reside within infected cells in lymph node tissue and peripheral blood and viral RNA sequences isolated from contemporaneous plasma we conducted phylogenetic analyses. Maximum likelihood phylogenetic trees of the single *gag*, *pro* and *pol* (p6 through nt 900 of RT) sequences isolated from memory and naïve CD4^+^ T-cells from the paired lymph node tissue, peripheral blood and plasma RNA samples from each of the five individuals were performed. Sequences from all five patient samples as well as standard laboratory viruses formed independent populations that were at least 5% different from one another, with no intermingling, demonstrating that the patient-specific viral populations were genetically distinct (data not shown).

**Figure 2:**
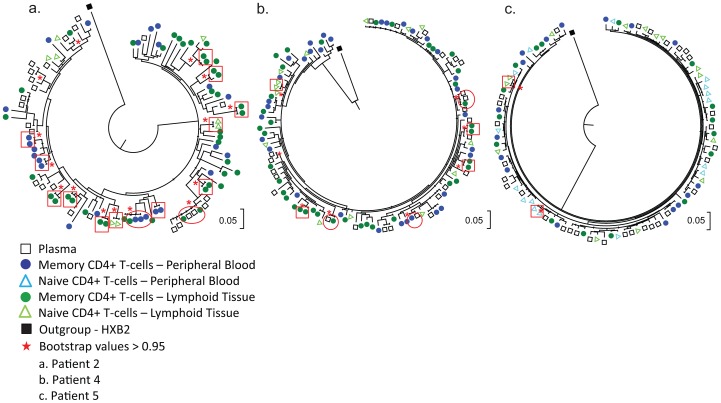
Phylogenetic analysis of sequences from the different viral compartments. Maximum likelihood trees of HIV-1 sequences isolated from memory and naïve T-cells from lymph node tissue (filled dark green circles and light green triangles), peripheral blood (filled dark blue circles and light blue triangles) and plasma viral RNA (black open squares). Red squares denote identical sequences from the same row and red circles denote identical sequences from different compartments. The trees represent sequences from patients (2 (a). 4 (b) and 5 (c)). Due to space limitations, the tree from patient 5 was made from reduced amount of sequences (randomly selected). For the original tree with all sequences included please see figure S1. All trees are rooted to HXB2 (filled black square).

Sequence analysis revealed that intracellular HIV-1 DNA in memory and naive CD4^+^ T-cells from lymph node tissue and peripheral blood samples and plasma RNA were phylogenetically similar in each of the patients (examples from patients 2, 4, and 5 are shown in **Fig. 2a–c**). In fact, we identified sequences in PBMC, lymphoid cells and plasma that were identical to each other (**Fig. 2** **red circles**, red squares indicate identical sequences from the same row). The genetic similarity of HIV-1 DNA populations in CD4^+^ T-cells from lymph node tissue, peripheral blood and HIV-1 RNA from plasma implies exchange of virus and/or infected cells between these compartments.

Genetic characterization of viral sequences within multiply infected cells (detected by two or more DNA molecules amplified from the same row of 10 wells) revealed both identical and genetically different HIV-1 variants within the same cell (**Table 3** **, columns 3–8**). For example, in memory CD4^+^ T-cells isolated from lymph node tissue we identified a total of 38 rows with doublet DNA molecules where 10 of the rows contained identical and 28 genetically different viral sequences. Identical HIV-1 DNA molecules detected in the same row could result from one cell being infected by two genetically identical virions, a cell in the process of S-phase DNA synthesis, or from multiple singly infected cells detected in the same row. It is highly unlikely that this finding was the result of two independently infected cells adhering to each other as 1) all the cells had been subjected to single cell sorting and formed a monodisperse population and 2) significantly greater frequencies of identical sequences were present in rows with more than one provirus (p<0.04 for all cell types, Fisher's exact test). Also, the frequency of identical sequences in memory cells in lymph nodes was about 3 fold higher than in blood, consistent with greater rates of either cell division or multiple infection, but the difference was not statistically significant. Although multiply infected cells were infrequent, sequence alignments from all patients showed clear presence of recombinant sequences which were identified by using a genetic algorithm for recombination detection (GARD, data not shown).

**Table 3:** Number of identical and distinct HIV-1 sequences.

1	2	3	4	5	6	7	8
**Observed number of HIV DNA molecules**							
**One**		**Two**		**Three**		**Four**	
**Lymphoid Tissue - Memory CD4+ T-cells**							
177		38		8		2	
**Identical**	**Distinct**	**Identical**	**Distinct**	**Identical**	**Distinct**	**Identical**	**Distinct**
17	160	10	28	3	5	1	1
**Lymphoid Tissue - Naive CD4+ T-cells**							
63		10		3		0	
**Identical**	**Distinct**	**Identical**	**Distinct**	**Identical**	**Distinct**	**Identical**	**Distinct**
8	55	4	6	1	2	0	0
**Peripheral Blood - Memory CD4+ T-cells**							
64		10		2		0	
**Identical**	**Distinct**	**Identical**	**Distinct**	**Identical**	**Distinct**	**Identical**	**Distinct**
2	62	5	5	0	2	0	0
**All cell types combined**							
304		58		13		2	
**Identical**	**Distinct**	**Identical**	**Distinct**	**Identical**	**Distinct**	**Identical**	**Distinct**
27	277	19	39	4	9	1	1

### Limited compartmentalization of HIV-1 between CD4^+^ T-cells in lymph node tissue, peripheral blood and plasma RNA

The degree of phylogenetic interspersion of sequences from different compartments in the trees (**Fig. 2a–c**) indicates genetic flow of HIV-1 between cells in lymph node tissue, peripheral blood and plasma during untreated chronic HIV-1 infection. To better understand the genetic relationship among the isolated sequences and assess whether the HIV-1 populations are structured by the different compartments from where they were isolated, we conducted compartmentalization tests using both distances-based (Wright's measure of population subdivision (Fst) [20]) and tree-based (the Slatkin Madison test [21] and Simmonds Association index [22](AI)) methods (**Table 4**). We first conducted compartmentalization analysis using the Wright's measure of population subdivision (Fst). As shown in Table 4, (column 7), nine of the 15 Fst analyses showed no statistical support for a genetic subdivision between sequences isolated from lymph node, peripheral blood or plasma. Statistical support for viral subdivision was, however, found in 6 of the Fst analyses, indicating a higher degree of structure than expected by chance. When conducting the Slatkin-Madison test, we found similar results with no statistical support for compartmentalization in 15 of 20 analyses and statistical evidence for compartmentalization in 5 of the 20 analyses (**Table 4** **, column 8**). The number of analyses that showed statistically significant evidence for compartmentalization did however drop to only 3 analyses for both the Fst and SM analyses after Bonferroni correction for multiple comparisons. This conclusion is consistent with limited evidence of compartmentalization as assessed by Simmonds Association Index (AI ranging from 0.45 to 1 with bootstrap values ranging from 0.30 to 1). An AI value of 1 implies that the clustering of HIV sequences does not deviate from that expected from random compartment association (randomness between sequences indicating no compartmentalization) while an AI value closer to 0 implies a structured compartmentalization between sequences. No patient except patient 2 showed evidence of compartmentalization in all three analyses conducted. However, the corresponding AI values indicate that the extent of compartmentalization in the samples from this patient is limited (**Table 4** **, column 10**). Identical sequences in the dataset can influence the results of the compartmentalization analyses. To evaluate this possibility, we collapsed all the identical sequences in each compartment into one sequence and repeated the analyses. As shown in **Table S1**, the amount of evidence for compartmentalization was clearly reduced when identical sequences were collapsed and we only found evidence for compartmentalization in all of the analyses in one of the datasets (peripheral blood vs plasma) for patient 2. Also, differences in the number of sequences in each compartment can influence the result of the compartmentalization analyses [23], therefore we conducted the same analyses based on an equal number of sequences in each compartment (**Table S2**), which did not lead to substantially different results. Taken together, these data suggest compartmentalization is infrequent during chronic HIV infection, and provide strong support for free HIV flow between lymphoid tissue and peripheral blood compartments.

**Table 4:** Evaluation of viral compartmentalization.

1	2	3	4	5	6	7	8	9	10	11
Patient	Analysis	Number of sequences				Fst	Slatkin Maddison		Simmonds Association	
		PB	LN	SGS	Total	P-value*	P-value*	Migration events°	AI†	Bootstrap*
1	PB/LN/SGS	**6**	**98**	**29**	**133**	N/A	0.1243	30	0.88	0.69
	PB/LN	**6**	**98**		**104**	0.88	1.0000	6	0.88	0.48
	PB/SGS	**6**		**29**	**35**	0.8923	1.0000	6	1.06	0.3
	LN/SGS		**98**	**29**	**127**	0.4123	***0.0459***	23	0.85	0.72
2	PB/LN/SGS	**25**	**49**	**25**	**99**	N/A	**0.0004** **	33	**0.58**	**1**
	PB/LN	**25**	**49**		**74**	***0.003*** **	***0.0292***	17	***0.51***	***1***
	PB/SGS	**25**		**25**	**50**	**0.001** **	**0.0002** **	9	**0.45**	**1**
	LN/SGS		**49**	**25**	**74**	***0.0056***	**0.0021** **	15	***0.54***	***0.99***
3	PB/LN/SGS	**32**	**95**	**27**	**154**	N/A	*0.0886*	50	0.89	0.83
	PB/LN	**32**	**95**		**127**	**0.0353**	*0.099*	26	0.78	0.92
	PB/SGS	**32**		**27**	**59**	0.0687	0.0561	16	0.77	0.84
	LN/SGS		**95**	**27**	**122**	0.7755	0.4475	24	0.95	0.51
4	PB/LN/SGS	**33**	**52**	**25**	**110**	N/A	0.3818	47	0.86	0.88
	PB/LN	**33**	**52**		**85**	0.1843	0.6823	27	0.88	0.68
	PB/SGS	**33**		**25**	**58**	0.1762	0.212	17	0.77	0.85
	LN/SGS		**52**	**25**	**77**	***0.0279***	0.1414	19	0.78	0.86
5	PB/LN/SGS	**57**	**74**	**31**	**162**	N/A	0.0913	66	***0.86***	***0.95***
	PB/LN	**57**	**74**		**131**	***0.002*** **	0.1036	39	0.81	0.9
	PB/SGS	**57**		**31**	**88**	0.0719	0.6033	26	0.89	0.66
	LN/SGS		**74**	**31**	**105**	0.5012	0.7898	28	0.89	0.68

* P values<0.05 (for Fst and SM) and bootstrap values>0.95 were considered statistically significant evidence of compartmentalization. P-values<0.05 and bootstrap values >0.95 are shown in bold and underlined.

** Statistically significant evidence for compartmentalization after Bonferroni correction for multiple comparisons.

° The number of migration events between the different populations in each phylogenetic tree.

† AI: Association index, where 0 indicates maximum phylogenetic structure and 1 indicates panmixia. Results in italic type are results that changed (either to no significance of compartmentalization or significance of compartmentalization) when running the same analyses with collapsed identical sequences or the same number of sequences from each compartment. N/A Not applicable.

### Similar genetic diversity in sequences isolated from CD4^+^ T-cells from lymph node tissue, peripheral blood and plasma RNA

To further evaluate the genetic relationship among sequences from the different cell types, cellular compartments and plasma, we measured average pairwise differences (APD) within each subset of sequences from each patient. In four of the five patients (patients 2–5) the genetic diversity was above one percent in each of the different compartments. However, the genetic diversity of the HIV population in patient 1 was lower, around 0.5% in the different compartments (**Table 5**). This difference probably reflects the shorter duration of infection in this patient compared to the other four [6], [24].

**Table 5:** Average pairwise distance.

1	2	3	4	5	6	7
Patient	Average Pairwise Distance (%)					
	LN		PBMC		Plasma	Overall
	Memory	Naive	Memory	Naive	Plasma	LN.PB.PL
1	0.6	0.2	0.5	N/A	0.4	0.5
2	2	1.7	1.7	N/A	1.7	1.9
3	1.3	1.3	1.6	N/A	1.5	1.4
4	1.2	1	1.2	N/A	1	1.1
5	1.5	1.5	1.6	1.4	1.5	1.5

The genetic diversity did not vary greatly within each cell type for all of the patients, averaging 1.3% (range = 0.6–2.0%) within the memory population from lymph node tissue and 1.1% (range 0.2–1.7%) in naïve T-cells from the same anatomical compartment (**Table 5** **, columns 2–3**). Similar diversity was found in sequences isolated from memory and naïve T-cells from peripheral blood, 1.3% (range = 0.5–1.7%) and 1.4% (data from patient 5 only) respectively (**Table 5** **, columns 4–5**). The APD of the sequences isolated from extracellular RNA was 1.2% (range = 0.4–1.7%, **Table 5** **, column 6**) which is similar to the cells analyzed from each compartment. Additional analysis did not indicate a statistically significant difference in average pairwise distance across the cellular subsets and plasma (p = 0.08, one-way repeated measures ANOVA). These results further indicate similar genetic composition of viral sequences isolated from memory and naïve T-cells from lymph node tissue, peripheral blood and viral sequences from plasma RNA in untreated chronic HIV-1 infection.

## Discussion

The rate of productive HIV-1 recombination is dependent on the frequency of cells infected with two or more genetically different HIV-1 variants. Multiple proviruses per infected cell have been reported in the spleen of some HIV-infected individuals [7]–[9]. We have recently shown, however, that the majority of CD4^+^ T-cells (>90%) in peripheral blood are not multiply infected [6]. The differences between our recent study and earlier studies may be attributable to the source of the cells (peripheral blood versus solid lymphoid tissue), since earlier studies have shown that most HIV-1 infection and viral replication takes place in lymphoid tissue such as lymph nodes and GALT [11]–[14]. The increased rate of HIV-1 infection and cell exposure to HIV-1 virions in lymphoid tissue could facilitate the transmission of multiple HIV-1 genomes to single cells, as reported for *in vitro* infections [25]. To investigate the difference in the frequency of multiply infected cells isolated from lymph node tissue and peripheral blood, we used the single-cell sequencing assay recently developed by our group which allowed us to quantify and genetically characterize the virus isolated from single-infected cells [6].

When we analyzed individual HIV-1 DNA molecules from memory and naïve CD4^+^ T-cells from paired lymph node and peripheral blood samples from five chronically untreated HIV-1-infected patients, we found that the majority (>90%) of both memory and naïve HIV-infected CD4^+^ T-cells in lymph nodes contained only one HIV-1 DNA molecule, and that the observed number of samples with multiple HIV-1 DNA molecules was close to that expected from the Poisson distribution (**Table 2**). In individual patient analyses, we did not detect evidence of multiple infection. Increasing the sample size by pooling the data from all of the patients did, however, reveal statistical support for multiple infection in memory CD4^+^ T-cells isolated from lymph node tissue (Table 2). Single cell sequencing revealed that some multiply infected cells had identical HIV-1 DNA sequences, which may arise from true multiple infection with HIV-1 with low genetic diversity (patient 1, low viral diversity) or from cells infected with a single provirus undergoing DNA replication. Restricting statistical analysis to data from patient 1 and only genetically distinct viruses of patients 2–5 was still suggestive of multiple infection, although the limited sample size and reduced statistical power yielded a more marginal p-value (0.066) to rule out 0% multiple infection. Overall, these findings suggest that levels of multiply infected cells with distinct proviruses are infrequent and our simulations predict that the percent of multiply infected cells is no greater than 7% in any of the cellular subsets analyzed (**Table 2** **, column 9**). Despite the low level of multiply infected cells, we did detect phylogenetic evidence of for recombination in all patient samples (data not shown) implying that only few multiply infected cells in peripheral blood and lymph node tissue are needed to generate recombinant viral variants. These results agree with our earlier studies of CD4^+^ T-cells from peripheral blood and with the results of Neher et al [26] and Batorsky et al [27], who used modelling based on the amount of viral recombination found during chronic HIV-1 infection, to show that only about 10% of HIV-1-infected cells are multiply infected. The role of low frequency multiply infected cells on the rate of HIV-1 spread or emergence of variants encoding multiple resistance mutations *in viv*o is unknown. Previous *in vitro* data indicates cell to cell transmission may facilitate HIV spread despite antiretroviral therapy [15], which can serve to generate new recombinant viruses. Quantifying the role of multiply infected cells in vivo will require additional analysis that models frequency of infected cells, replication rates, as well as the size of replicating populations. Our estimates of the frequency of multiply infected cells are substantially lower than that reported previously [7], [8]. The reasons for the discrepancy between our and previously reported results are uncertain; it is possible that the source of cells isolated for analysis (spleen [7], [8] vs. PBMC, inguinal, and axillary lymph node [6, present ms.]) are responsible, in part, for the differences. Functional differences between these lymphoid organs may also play a role; immune cells, such as dendritic cells, which concentrate HIV-1 on their cell surface and facilitate infection of T-cells, may be essential participants in presenting genetically distinct HIV-1 to susceptible cells but differentially distributed in spleen and lymph node of HIV-1 infected individuals. Hence, one possible explanation for differences in numbers of infected cells may be higher DC – T-cell interactions in the spleen compared to other lymphoid node structures. It is unlikely that differences in clinical status of source patients was responsible for differences. Gratton et al. [7] and Jung et al. [8] studied patient with AIDS; two of our patients also met AIDS criteria (Table 1) and we did not detect substantial differences in numbers of multiply infected cells in AIDS and non-AIDS patients. In addition, the frequency of multiply infected cells was fairly constant across the majority of the patients studied, and did not correlate with frequency of infected cells, or with level of plasma RNA; similar levels of multiply infected cells were present in patients with viral RNA levels of 10^3^ or 10^5^ copies/ml. These data suggest that, *in vivo*, multiple infection events of cells from lymph node tissue or peripheral blood are not determined by levels of circulating HIV-1 and other mechanisms may facilitate multiple infection.

Our studies revealed that the average infection frequency of lymph node derived memory CD4^+^ T-cells (250 cells per HIV DNA molecule, 0.4%) was 2–17 times higher but not significantly different from peripheral blood derived memory CD4^+^ T-cells (700 cells per HIV DNA molecule, 0.14%). The higher HIV-1 infection frequency of cells from lymph node tissue compared to peripheral blood is consistent with the higher concentration of HIV-1 in lymphoid tissue [11], [28],[29]. In both peripheral blood and lymph node we found that the infection frequency of memory CD4^+^ T-cells was substantially higher than that of naïve CD4^+^ T-cells. This difference in infection frequency has been explained by the expression of the CCR5 co-receptor in memory T-cells compared to naïve T-cells [30], [31]. In addition, the frequency of infection of one cell type compared to another has been related to the number of proviruses per infected cell [16]. Since the majority (>90%) of memory and naïve T-cells from peripheral blood and lymph node tissue contain only one HIV-1 DNA molecule, the higher infection frequency of memory compared to naïve T-cells is not related to differences in the number of HIV-1 DNA molecules within each infected cell. We also found that the major component of the viral reservoir in both peripheral blood and lymph node tissue was composed of memory T-cells in peripheral blood and lymph node tissue. The contribution of non-T cells to dual infection is uncertain; previously we did not detect infected monocytes in the periphery, it is not known whether non T-cells in tissues sources (e.g., macrophages microglial cells) may contribute to dual infection. Given the relatively high contribution of memory CD4^+^ T-cells in lymph node tissue and the fact that most of the CD4^+^ T-cells are located in lymphoid tissue these data suggest that the major HIV-1 reservoir is located in memory CD4^+^ T-cells residing in lymphoid tissue consistent with previous data [32], [33]. Direct comparative analyses revealed that 80–97% of infected memory cells and 82–100% of infected naïve cells were present in lymph nodes. Reasons for variability in distribution are uncertain, but may be related to duration of infection and degree of immunodeficiency. It was unlikely that lymph node “exhaustion” was responsible for variability; all of the lymph nodes were enlarged, had clear histologic follicular hyperplastic architecture without obvious fibrosis, and yielded large numbers (1×10^9^–8×10^10^) of cells for analysis. We also found a strong correlation between levels of plasma RNA and frequency of infected memory cells in lymphoid-derived material (r^2^ = 0.77 and 0.79, methods 1 and 2, respectively). Consistent with previous observations of Brenchley and coworkers [16] we also found a correlation between plasma HIV RNA and frequency of infected memory cells in peripheral blood.

The viral genetic makeup of multiple infected cells is important for the production of heterodimeric virions and the development of new recombinant variants. Using single-cell sequencing, in four of the five patients we were able to detect more than one HIV-1 DNA molecule per cell in memory and naïve CD4^+^ T-cells from lymph node tissue and memory T-cells from peripheral blood. These molecules could be the result of multiply infected cells. When analyzing the genetic makeup of the virus within these infected cells we found both identical and distinct HIV-1 variants in single rows of cellular lysate. In total we found 49 rows (all patients and cell types combined) with distinct viral variants, which, if they arose from multiple infection could give rise to heterodimeric virions and new viral recombinants. One drawback of the SCS assay is however that we cannot distinguish whether the HIV-1 DNA that we are analyzing is in an integrated, linear, or circular form or productive or not. The presence of un-integrated HIV-1 DNA has been well documented [34], [35] hence, even though we detect different proviruses in the same lane we cannot determine if these possible multiply infected cells will produce virions with two different RNA molecules or not.

We also detected 10 individual cells in both the memory and naïve cell compartments with at least 2 identical sequences. The presence of identical HIV-1 sequences within a single cell suggests a scenario in which the cell had been infected, and subsequently underwent DNA replication. Alternatively, such cells could have been infected by cell-cell transfer of genetically identical virions from a nearby cell. Both infected memory and naïve CD4 cells have been reported to be capable of proliferation in HIV infected individuals [36], [37], and the greater extent of proliferation of these cells in lymph nodes than in blood is consistent with the difference in frequency of identical HIV sequences in the two compartments.

Determining the genetic relationship among HIV-1 populations from peripheral blood and tissue compartments is important for understanding the dynamics of HIV-1 infection. Using single-cell sequencing we demonstrated that the intracellular HIV-1 DNA sequences isolated from memory and naïve CD4^+^ T-cells residing in lymph node tissue are phylogenetically interspersed with intracellular sequences from cells in peripheral blood and plasma-derived RNA sequences. The similarity of sequences from lymph node tissue, peripheral blood, and plasma was confirmed by two phylogenetic approaches, maximum likelihood tree construction (intermingling of sequences) and compartmentalization tests (lack of signal for compartmentalization in 4 of the 5 patients). We did find statistical support for compartmentalization by all three compartmentalization tests (Wrights measurement of subdivision, Simmonds Association Index and the Slatkin-Madison) in samples from patient 2. However, the corresponding AI values indicate that the extent of compartmentalization is limited. Also, the statistically significant evidence for compartmentalization was clearly reduced after Bonferroni correction for multiple comparisons and when identical sequences from the same compartment were collapsed. These results indicate that there is considerable flow of virus and/or infected cells between lymph node tissue and peripheral blood and that the peripheral blood reflects the genetic make-up of HIV-1 in lymph node tissue of untreated patients. The fact that we find the same frequency of multiply infected memory CD4^+^ T-cells in lymph node tissue (7%) as in peripheral blood (6%) also indicates that HIV-1 infected cells are trafficking between these two compartments. In addition, we found genetically identical sequences from plasma-derived RNA and memory CD4^+^ T-cells from both peripheral blood and lymph node tissue. This observation could be explained by circulation of viral variants or infected cells between the lymph node tissue and peripheral blood compartments.

In conclusion, by using the single-cell sequencing assay we found that the majority (>90%) of memory and naïve CD4^+^ T-cells isolated from both lymph node tissue and peripheral blood contain only one HIV-1 DNA molecule. We show a higher infection frequency in memory CD4^+^ T-cells compared to naïve T-cells from lymph node tissue. The genetic similarity of HIV-1 DNA populations in CD4^+^ T-cells from lymph node tissue, peripheral blood and HIV-1 RNA from plasma implies ongoing exchange of virus and/or infected cells between these compartments during untreated chronic HIV-1 infection.

## Materials and Methods

### Ethical statement

Written informed consent was provided by all study participants. The study was approved by the institutional review boards at the NIH and the Karolinska Institutet.

### Patients

Participants in this study were chronically infected with HIV-1, subtype B (all Feibig stage VI), were not receiving antiretroviral therapy, and were enrolled in clinical studies at National Institute of Allergy and Infectious Disease (NIAID) Critical Care Medical Department (NIAID/CCMD) Clinic of National Institutes of Health (NIH), Bethesda, Maryland (**Table 1**). One patient had received transient antiretroviral therapy but had not undergone any therapy for over two years prior to biopsy; the remaining four patients were antiretroviral naïve. Two patients (3,4) had CD4 percent <14% and therefore have AIDS. Palpable lymph nodes were identified in axilla (2) or inguinal (3) regions and entire lymph nodes were excised. In one case (patient 3), the node was removed because of a suspicion of tuberculosis. Nodal tissue was divided for research and standard histopathology; in all cases, review of histopathology revealed preserved nodal architecture with marked follicular hyperplasia and plasmacytosis characteristic of HIV lymphadenopathy. One participant (patient 3) had necrotizing granulomas present, with no pathogens identified by staining, culture, or PCR amplification. All participants tolerated the procedure well; one participant had persistent seroma, which was treated with antibiotic therapy for possible infection, and which resolved completely. Peripheral blood samples were obtained at the time of, or within 3 weeks of biopsy.

### Single-cell sequencing

The single-cell sequencing assay [6] was used to quantify and genetically analyze the intracellular HIV-1 viral populations found in memory and naïve CD4^+^ T cells from lymph node tissue and peripheral blood. In brief, pools of cells, each containing <<1 infected cell*, were lysed and distributed across 10 wells per row in a total of 8 rows on a 96 well PCR plate. PCR amplification (p6 through nt 1–900 of RT) and sequencing of the DNA in each well allowed enumeration and analysis of the genetic relationship of viral DNA molecules in each infected cell. For a detailed description we refer to the original publication [6].

*To determine the concentration of cells containing less than one infected cell per well, several plates were set up with different amount of cells sorted into each well ranging from 30–1000 cells/well. A test run for each cellular concentration was performed and from the number of HIV-1 DNA molecules amplified by PCR in the different test runs the frequency of infection for each sorted cell concentration was calculated for each patient to determine the concentration level of cells containing far less than one HIV-1-infected cell. On the basis of these calculations, additional HIV-1 DNA amplification PCR plates were set up for the particular sorted cell concentration determined to contain much less than one HIV-1–infected cell.

The SCS was used as previously described with minor changes described below.

### Isolation of PBMCs and single cells from lymph node tissue

Lymph node biopsy samples were processed directly after excision. Single cell suspensions were obtained by gentle mechanical manipulation of the lymph node tissue [38], [39]. Blood samples from the five patients were collected and peripheral blood mononuclear cells (PBMCs) were separated from the plasma using Ficoll.

### Fluorescence activated cell sorting

Cryopreserved cells from lymph node tissue and peripheral blood were thawed in R10 media (RPMI medium 1640, 10% fetal bovine serum (FBS), 100 U/mL penicillin, 100 µg/mL streptavidin, 2 mM L-glutamine and 20 mM HEPES) at 37°C, immediately washed twice with R10 and then rested in R10 containing 20 U of DNase/ml (Roche) for 1 hour. Cells were then spun down and washed with Dulbecco' s phosphate buffered saline (Gibco). Cells were spun down once more and resuspended in a minimal volume and then stained with LIVE/DEAD Violet Viability/Vitality stain (Invitrogen). Ten minutes after the addition of LIVE/DEAD Violet Viability/Vitality stain pre-titered amounts of CD3 H7APC, CD4 Cy55PE, CD8 APC, CD45RO TRPE, CD27 Cy5PE and CD14 Pacific Blue and CD19 Pacific Blue were added to the cells, which were subsequently incubated for an additional 20 minutes at room temperature. Cells were washed once with R10 and then immediately sorted into 96 well plates using a modified FACSAria flow cytometer at 70psi. Both peripheral blood cells and cells from lymph nodes were sorted in the same manner. Singlet cells were sorted based on Forward Scatter Height (FSC-H) and Forward Scatter Area (FSC-A). Dead cells, B cells, and monocytes were excluded by staining with either LIVE/DEAD fixable violet dead cell stain or CD14 and CD19 staining. Naive (CD27+CD45RO−) and memory (CD27+/CD45RO+ and CD45RO+/−CD27−) CD3+CD4+CD8− small lymphocytes were then sorted in 96 well PCR plates containing 50 µl of lysis buffer.

### Antibodies

CD3 H7APC and CD8 APC were purchased from BD Bioscience. CD4 Cy55PE, CD14 and CD19 Pacific Blue were from Invitrogen. CD45 RO TRPE and CD27 Cy5PE were from Beckman Coulter.

### Single genome sequencing

To compare the intracellular populations identified using the single-cell sequencing assay to HIV-1 RNA populations found in plasma, we performed single-genome sequencing (SGS) on the plasma samples from each of the 5 patients as described earlier [24], [40], [41]. The viral region amplified from plasma spanned from p6 through RT and was the same region that was amplified from intracellular viral DNA.

### Phylogenetic analyses

Alignments of the intracellular and extracellular HIV-1 populations were performed using an in-house computer program written in Perl scripting language (available upon request). Recombination was screened using a genetic algorithm for recombination detection (GARD) [42]. For phylogenetic analysis of the HIV-1 populations, maximum likelihood (ML) phylogenetic trees were constructed in PhyML version 3.0 [43]. We used the general time reversible (GTR) nucleotide substitution model incorporating gamma-distributed rate variation among sites and allowing a proportion of invariable sites. Branch support was inferred using 1000 bootstrap replicates. All sequences obtained from the five patients were compared by phylogenetic analysis to each other and standard laboratory viruses to ensure that no contamination between patient samples or lab strains had occurred. Evidence for compartmentalization between sequences from lymph node, peripheral blood and plasma was evaluated using tree-based methods, namely Simmond's Association Index (AI) [22] and the Slatkin-Maddion (SM) test [21]. AI statistical support was obtained using 1000 bootstrap trees and 10 re-labelings per sample (only bootstrap values above 0.95 were considered significant). For the SM method, 10000 permutations were performed (p-values<0.05 were considered significant). In addition, we used the nucleotide distance-based statistic known as Wright's measure of population subdivision (F_ST_) [20]. Distances were estimated using a ML approach under a GTR nucleotide substitution model, estimating all parameters independently for each branch. To obtain the significance of the statistics, 10000 permutations were computed, with the permutation test randomly allocating sequences into lymph node, peripheral blood or plasma pre-defined clades. All compartmentalization tests were performed using the package Hyphy [44]. Measurements of the HIV-1 genetic diversity (average pairwise distance, APD) within each cell type and plasma were calculated using MEGA5.0 (http://www.megasoftware.net/). Diversities are reported as percent differences.

### Calculations and statistical methods

Rates of infected CD4+ T-cells, rates of multiple infection, and comparisons thereof were calculated using methods previously described [6].

We estimated total number of infected cells in the blood compartment by determining total blood volume using the Nadler formula [45]. Total infected cell levels in lymphoid node were calculated using proportion of lymph node used for analysis, total yield of cells, and reported estimates of the number of lymph nodes in humans [46].

## Supporting Information

Figure S1 **Phylogenetic analysis of sequences from the different viral compartments: patient 5.** Maximum likelihood trees of HIV-1 sequences isolated from memory and naïve T-cells from lymph node tissue (filled dark green and light green circles), peripheral blood (filled dark blue and light blue circles) and plasma viral RNA (black open squares). Red squares denote identical sequences from the same row and red circles denote identical sequences from different compartments. The tree is rooted to HXB2 (filled black square).
(EPS)

Table S1 **Evaluation of viral compartmentalization after collapsing identical sequences in each compartment.**
(DOCX)

Table S2 **Evaluation of viral compartmentalization with equal number of sequences in each compartment.**
(DOCX)
