# Supplementary material for: Single Cell Analysis of Lymph Node Tissue from HIV-1 Infected Patients Reveals that the Majority of CD4+ T-cells Contain One HIV-1 DNA Molecule
Source: PLoS Pathog. 2013 Jun 20;9(6):e1003432. doi: 10.1371/journal.ppat.1003432 (PMC3688524; doi:10.1371/journal.ppat.1003432)
Supplement: Table S1 — Evaluation of viral compartmentalization after collapsing identical sequences in each compartment. (DOCX) [file ppat.1003432.s002.docx]

**Table S1. Evaluation of viral compartmentalization after collapsing identical sequences in each compartment.**

| **1** | **2** | **3** | **4** | **5** | **6** | **7** | **8** | **9** | **10** | **11** |  |
| --- | --- | --- | --- | --- | --- | --- | --- | --- | --- | --- | --- |
| **Patient** | **Analysis** | **Number of sequences** | | | | **Fst** | **Slatkin Maddison** | | **Simmonds Association** | |  |
|  |  | **PB** | **LN** | **SGS** | **Total** | **P-value*** | **P-value*** | **Migration events⁰** | **AI†** | **Bootstrap*** |  |
| **1** | PB/LN/SGS | **6** | **77** | **25** | **108** | N/A | 0.205 | 26 | 0.90 | 0.614 |  |
|  | PB/LN | **6** | **77** |  | **83** | 0.8983 | 1.0000 | 6 | 0.95 | 0.429 |  |
|  | PB/SGS | **6** |  | **25** | **31** | 0.6607 | 1.0000 | 6 | 1.00 | 0.335 |  |
|  | LN/SGS |  | **77** | **25** | **102** | 0.9341 | 0.1582 | 20 | 0.87 | 0.67 |  |
| **2** | PB/LN/SGS | **21** | **42** | **23** | **86** | N/A | **0.0208** | 32 | 0.77 | **0.989** |  |
|  | PB/LN | **21** | **42** |  | **63** | 0.7648 | 0.202 | 16 | 0.74 | 0.885 |  |
|  | PB/SGS | **21** |  | **23** | **44** | **0.0106** | **<0.001**** | 8 | 0.59 | **0.984** |  |
|  | LN/SGS |  | **42** | **23** | **65** | 0.1595 | **0.0219** | 15 | 0.71 | 0.941 |  |
| **3** | PB/LN/SGS | **30** | **90** | **27** | **147** | N/A | 0.1711 | 49 | 0.96 | 0.518 |  |
|  | PB/LN | **30** | **90** |  | **120** | **0.001**** | 0.6096 | 27 | 0.87 | 0.713 |  |
|  | PB/SGS | **32** |  | **27** | **59** | 0.0641 | 0.0921 | 16 | 0.82 | 0.752 |  |
|  | LN/SGS |  | **90** | **27** | **117** | 0.344 | 0.256 | 23 | 0.96 | 0.518 |  |
| **4** | PB/LN/SGS | **33** | **49** | **24** | **106** | N/A | 0.5831 | 47 | 0.96 | 0.53 |  |
|  | PB/LN | **33** | **49** |  | **82** | 0.1965 | 0.7446 | 27 | 0.95 | 0.471 |  |
|  | PB/SGS | **33** |  | **24** | **57** | 0.4636 | 0.2882 | 17 | 0.82 | 0.73 |  |
|  | LN/SGS |  | **49** | **24** | **73** | 0.549 | 0.4873 | 20 | 0.90 | 0.597 |  |
| **5** | PB/LN/SGS | **55** | **69** | **31** | **155** | N/A | 0.2048 | 65 | 0.95 | 0.627 |  |
|  | PB/LN | **55** | **69** |  | **124** | 0.1831 | 0.4506 | 40 | 0.92 | 0.612 |  |
|  | PB/SGS | **55** |  | **31** | **86** | 0.8302 | 0.4569 | 25 | 0.93 | 0.53 |  |
|  | LN/SGS |  | **69** | **31** | **100** | 0.6441 | 0.6379 | 27 | 0.95 | 0.477 |  |
| * P values < 0.05 (for Fst and SM) and bootstrap values > 0.95 were considered statistically significant evidence of compartmentalization. | | | | | | | | | | | |
| P-values <0.05 and bootstrap values >0.95 are shown in bold and underlined. | | | | | | | | | | | |
| ** Statistically significant evidence for compartmentalization after Bonferroni correction for multiple comparisons. | | | | | | | | | | | |
| ⁰ The number of migration events between the different populations in each phylogenetic tree. | | | | | | | | | | | |
| † AI: Association index. where 0 indicates maximum phylogenetic structure and 1 indicates panmixia. | | | | | | | | | | | |
| N/A Not applicable | | | | | | | | | | | |
