# Supplementary material for: Single Cell Analysis of Lymph Node Tissue from HIV-1 Infected Patients Reveals that the Majority of CD4+ T-cells Contain One HIV-1 DNA Molecule
Source: PLoS Pathog. 2013 Jun 20;9(6):e1003432. doi: 10.1371/journal.ppat.1003432 (PMC3688524; doi:10.1371/journal.ppat.1003432)
Supplement: Table S2 — Evaluation of viral compartmentalization with equal number of sequences in each compartment. (DOCX) [file ppat.1003432.s003.docx]

**Table S2. Evaluation of viral compartmentalization with equal number of sequences in each compartment.**

| **Patient** | **Analysis** | **Number of sequences‡** | | | | **Fst** | **Slatkin Maddison** | | **Simmonds Association** | |  |
| --- | --- | --- | --- | --- | --- | --- | --- | --- | --- | --- | --- |
|  |  | **PB** | **LN** | **SGS** | **Total** | **P-value*** | **P-value*** | **Migration events⁰** | **AI†** | **Bootstrap*** |  |
| **1** | PB/LN/SGS | **6** | **6** | **6** | **18** | N/A | 0.9294 | 10 | 1.09 | 0.23 |  |
|  | PB/LN | **6** | **6** |  | **12** | 0.3607 | 0.9308 | 5 | 0.93 | 0.35 |  |
|  | PB/SGS | **6** |  | **6** | **12** | 0.2668 | 0.5848 | 4 | 1.24 | 0.15 |  |
|  | LN/SGS |  | **6** | **6** | **12** | 0.9714 | 0.9294 | 5 | 1.08 | 0.26 |  |
| **2** | PB/LN/SGS | **25** | **25** | **25** | **75** | N/A | **<0.001**** | 26 | **0.65** | **0.99** |  |
|  | PB/LN | **25** | **25** |  | **50** | **0.0224** | 0.1011 | 14 | **0.67** | **0.96** |  |
|  | PB/SGS | **25** |  | **25** | **50** | **0.0007**** | **0.0002**** | 9 | **0.45** | **1** |  |
|  | LN/SGS |  | **25** | **25** | **50** | 0.081 | **0.0032** | 11 | 0.71 | 0.93 |  |
| **3** | PB/LN/SGS | **27** | **27** | **27** | **81** | N/A | **0.0522** | 34 | 0.86 | 0.85 |  |
|  | PB/LN | **27** | **27** |  | **54** | **0.0117** | **0.0083** | 13 | 0.70 | 0.93 |  |
|  | PB/SGS | **27** |  | **27** | **54** | 0.0732 | 0.0849 | 15 | 0.78 | 0.82 |  |
|  | LN/SGS |  | **27** | **27** | **54** | 0.8043 | 0.1853 | 16 | 1.00 | 0.38 |  |
| **4** | PB/LN/SGS | **25** | **25** | **25** | **75** | N/A | 0.2844 | 34 | 0.82 | 0.91 |  |
|  | PB/LN | **25** | **25** |  | **50** | 0.1593 | 0.2096 | 15 | 0.81 | 0.72 |  |
|  | PB/SGS | **25** |  | **25** | **50** | 0.2168 | 0.3871 | 16 | 0.76 | 0.84 |  |
|  | LN/SGS |  | **25** | **25** | **50** | 0.1111 | 0.3994 | 16 | 0.80 | 0.78 |  |
| **5** | PB/LN/SGS | **31** | **31** | **31** | **93** | N/A | 0.3017 | 43 | 0.90 | 0.76 |  |
|  | PB/LN | **31** | **31** |  | **62** | 0.1466 | 0.9371 | 24 | 0.87 | 0.64 |  |
|  | PB/SGS | **31** |  | **31** | **62** | 0.4277 | 0.8407 | 23 | 0.98 | 0.37 |  |
|  | LN/SGS |  | **31** | **31** | **62** | 0.558 | 0.1931 | 19 | 0.89 | 0.62 |  |
| ‡ Sequences were collected randomly to create groups with the same amount of sequences. | | | | | | | | | | | |
| * P values < 0.05 (for Fst and SM) and bootstrap values > 0.95 were considered statistically significant evidence of compartmentalization. | | | | | | | | | | | |
| P-values <0.05 and bootstrap values >0.95 are shown in bold and underlined. | | | | | | | | | | | |
| ** Statistically significant evidence for compartmentalization after Bonferroni correction for multiple comparisons. | | | | | | | | | | | |
| ⁰The number of migration events between the different populations in each phylogenetic tree. | | | | | | | | | | | |
| † AI: Association index. where 0 indicates maximum phylogenetic structure and 1 indicates panmixia. | | | | | | | | | | | |
| N/A Not applicable | | | | | | | | | | | |
